# A Sulfated Acidic Heteropolysaccharide from Sea Cucumber Cooking Liquid Suppresses HCT-15 Colorectal Cancer Cell Proliferation by Inducing ROS-Associated Mitochondrial Apoptosis and DNA Damage Response

**DOI:** 10.3390/md24080270

**Published:** 2026-08-04

**Authors:** Xiaoxiao Liu, Shengquan Xu, Ruoxi Sun, Binzhuo Liu, Peng Peng, Kairui Feng

**Affiliations:** 1 School of Pharmacy, Shandong Second Medical University, Weifang 261053, China; 2 School of Pharmacy, East China Normal University, Shanghai 200241, China

**Keywords:** sea cucumber cooking liquid, sulfated acidic heteropolysaccharide, P0.7, colorectal cancer, DNA damage response

## Abstract

Sea cucumber cooking liquid contains water-soluble macromolecules, but its bioactive polysaccharide fractions remain insufficiently characterized. In this study, a polysaccharide-rich fraction, P0.7, was isolated from sea cucumber cooking liquid and evaluated for its antitumor activity against HCT-15 colorectal cancer. P0.7 was characterized as a relatively homogeneous sulfated acidic heteropolysaccharide-rich fraction containing 83.61 ± 3.65% total sugar, 13.67 ± 2.48% sulfate, 9.98 ± 1.22% uronic acid, and 5.11 ± 0.34% protein. It was mainly composed of galactose, mannose, and glucose, accounting for 34.12%, 26.93%, and 17.99%, respectively. Among the tested tumor cell lines, HCT-15 cells showed the highest sensitivity to P0.7, with inhibition rates of approximately 45% and 63% at 100 and 200 μg/mL, respectively. P0.7 promoted apoptosis, induced G2/M-phase accumulation, increased ROS production, disrupted mitochondrial membrane potential, regulated Bax, Bcl-2, and cleaved caspase-3 expression, and enhanced γ-H2AX-related DNA damage-response signaling in HCT-15 cells. In an HCT-15 xenograft mouse model, P0.7 reduced terminal tumor volume and tumor weight without causing obvious body weight loss. Histological and immunohistochemical analyses further showed reduced Ki67 staining, increased TUNEL-positive signals, and enhanced γ-H2AX staining in tumor tissues. These findings indicate that P0.7 suppresses HCT-15 colorectal cancer growth in vitro and in vivo, possibly through mechanisms associated with ROS accumulation, mitochondrial apoptosis, and γ-H2AX-related DNA damage response. These findings provide additional experimental evidence supporting the investigation of sea cucumber-derived polysaccharides for potential pharmaceutical applications.

## 1. Introduction

Colorectal cancer remains a major global health burden and is among the leading causes of cancer-related morbidity and mortality worldwide [1]. Surgery, chemotherapy, radiotherapy, targeted therapy, and immunotherapy have improved the management of selected patients. However, treatment resistance, cumulative toxicity, tumor recurrence, and heterogeneous therapeutic responses continue to limit long-term benefit, particularly in advanced disease [2,3]. These limitations support continued investigation of structurally defined natural products and marine-derived macromolecules as complementary sources for antitumor research.

Marine-derived compounds have emerged as an important source of anticancer leads because they encompass structurally diverse metabolites and macromolecules with mechanisms of action that are often distinct from those of terrestrial natural products [4,5]. Marine bioactives, including polysaccharides, peptides, alkaloids, terpenoids, and cyclic depsipeptides, have been reported to inhibit tumor growth through apoptosis induction, cell cycle disruption, angiogenesis inhibition, metastasis suppression, and modulation of oncogenic signaling pathways [6]. The preclinical and clinical development of marine-derived agents further demonstrates the translational potential of marine resources for the discovery of effective antitumor therapeutics.

Natural polysaccharides constitute a diverse class of bioactive macromolecules with potential antitumor activities [7,8,9]. Their biological properties are influenced by molecular weight, monosaccharide composition, glycosidic linkage, branching pattern, uronic acid content, sulfate substitution, and chain conformation [10]. In particular, acidic residues and sulfate groups affect charge density and may modify interactions with cellular surfaces, extracellular proteins, and signaling-associated biomolecules [11]. Accordingly, the purification and characterization of defined polysaccharide fractions are essential for establishing a reliable material basis for biological evaluation.

Marine polysaccharides are of particular interest because their structural diversity frequently includes sulfation and other anionic features that are associated with a broad range of biological effects [12]. Sea cucumbers contain structurally distinctive sulfated polysaccharides, principally fucosylated chondroitin sulfates and sulfated fucans [13,14]. Their physicochemical properties and biological activities vary with species origin, extraction conditions, molecular size, monosaccharide composition, and sulfation pattern [15,16]. Although these materials have been extensively investigated in sea cucumber tissues, water-soluble polysaccharide fractions released during industrial thermal processing have received substantially less attention.

Sea cucumber cooking liquid is generated during thermal processing and contains soluble macromolecules released from the raw material. Early work demonstrated the feasibility of recovering polysaccharides and related components from sea cucumber processing waste liquid [17,18]. More recent studies have reported antioxidant properties of cooking liquid polysaccharides, metabolic and gut microbiota-related effects of sulfated cooking liquid polysaccharides, and the structural and anti-*Helicobacter pylori* properties of a fucose-rich fucoidan isolated from this matrix [18,19,20,21,22]. A recent cell-based study further indicated that sea cucumber cooking liquid polysaccharides can mitigate H_2_O_2_-induced oxidative injury in RAW264.7 macrophages [23]. Although antitumor activities have been reported for sulfated polysaccharides derived from sea cucumber tissues, comparable evidence for chromatographically purified polysaccharide fractions recovered from sea cucumber cooking liquid remains limited. Collectively, these studies support the value of cooking liquid as a recoverable marine resource; however, most available work has emphasized crude preparations, fucose-rich fractions, or non-tumor-related activities. The present study advances previous work by combining the purification and characterization of a compositionally distinctive cooking liquid-derived fraction with cellular evaluation and in vivo antitumor validation.

In the present study, a previously undescribed polysaccharide-rich fraction, designated P0.7 according to its elution with 0.7 mol/L NaCl, was isolated for the first time from sea cucumber cooking liquid and characterized as a low-molecular-weight sulfated acidic heteropolysaccharide-rich fraction. A panel of established tumor cell lines from different tissue origins was initially evaluated to provide a preliminary comparison of their responsiveness to P0.7. HCT-15 cells were selected for subsequent mechanistic studies because they showed the highest sensitivity in this screening. Its antitumor effect was further examined in an HCT-15 xenograft model. By integrating chromatographic purification, physicochemical characterization, cellular mechanistic evaluation, and in vivo validation, this study aimed to clarify the biological potential of P0.7 and to provide experimental support for further pharmaceutical research on bioactive polysaccharides derived from sea cucumber processing resources.

## 2. Results

### 2.1. Structural Characterization of P0.7

**Figure 1:**
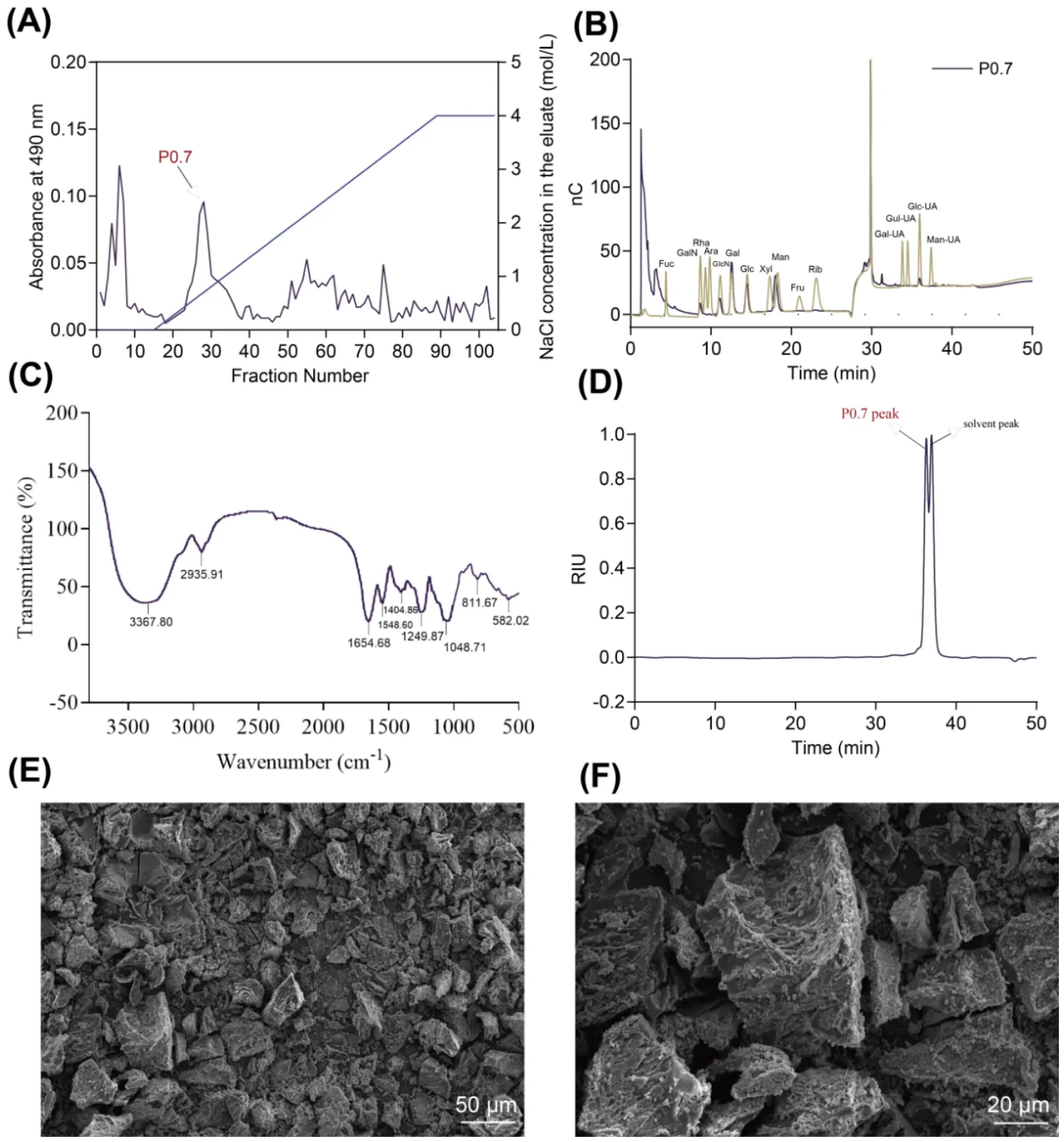
Purification and physicochemical characterization of P0.7 isolated from sea cucumber cooking liquid. (**A**) Elution profile of P0.7 during column chromatographic purification, monitored by the phenol sulfuric acid method at 490 nm. The NaCl gradient is shown as a blue line. (**B**) Monosaccharide composition analysis of P0.7 by chromatographic profiling using monosaccharide standards. (**C**) FT-IR spectrum of P0.7. (**D**) RI chromatogram of P0.7 showing a predominant polysaccharide peak and a solvent peak. (**E**,**F**) SEM images showing the dried-state morphology of P0.7. Scale bars = 50 μm in (**E**) and 20 μm in (**F**).

P0.7 was isolated from sea cucumber cooking liquid by column chromatography and subsequently subjected to physicochemical and spectroscopic characterization. The elution profile showed a distinct carbohydrate containing fraction during NaCl gradient elution, which was collected as P0.7 (Figure 1A). The yield of P0.7 from the crude polysaccharide fraction was 24.22%, indicating that P0.7 represented a major recoverable polysaccharide fraction from sea cucumber cooking liquid. Refractive index (RI) chromatographic analysis showed that P0.7 eluted as a predominant polysaccharide peak that was clearly separated from the solvent peak (Figure 1D), indicating that P0.7 was a relatively homogeneous polysaccharide-rich fraction. Molecular weight analysis further showed that P0.7 had a relatively low molecular weight of approximately 6.7 kDa. These results indicate that P0.7 was effectively enriched from sea cucumber cooking liquid and represented a relatively homogeneous polysaccharide-rich fraction.

#### 2.1.1. Physicochemical Properties of P0.7

The chemical composition of P0.7 was determined to define its basic physicochemical properties. P0.7 contained 83.61 ± 3.65% total sugar, 5.11 ± 0.34% protein, 13.67 ± 2.48% sulfate, and 9.98 ± 1.22% uronic acid. The high sugar content indicated that saccharides were the major components of P0.7, whereas the detectable sulfate and uronic acid contents demonstrated that P0.7 possessed typical features of an acidic sulfated polysaccharide. The relatively low but measurable protein content indicated the presence of minor proteinaceous components in the purified fraction.

Monosaccharide composition analysis showed that P0.7 consisted of fucose, galactosamine, glucosamine, galactose, glucose, mannose, ribose, and glucuronic acid, with molar percentages of 2.96%, 4.37%, 8.75%, 34.12%, 17.99%, 26.93%, 1.44%, and 3.44%, respectively (Figure 1B). Among these monosaccharides, galactose, mannose, and glucose were the predominant components, together accounting for approximately 79.04% of the total monosaccharide composition. The presence of glucuronic acid and amino sugars, together with the sulfate content, further supported the acidic and sulfated nature of P0.7. Based on these compositional features, P0.7 was characterized as a sulfated acidic heteropolysaccharide-rich fraction rather than a simple neutral polysaccharide.

The surface morphology of P0.7 was examined by scanning electron microscopy (SEM). In the dried state, P0.7 appeared as irregular fragments and sheet-like aggregates with a rough and folded surface (Figure 1E,F). This morphology indicated that P0.7 formed aggregated structures after drying, which may be associated with intermolecular interactions among abundant hydroxyl, carboxyl, and sulfate groups.

#### 2.1.2. FT-IR and NMR Spectroscopic Characterization of P0.7

FT-IR spectroscopy was performed to identify the major functional groups of P0.7. As shown in Figure 1C, the broad absorption band at 3367.80 cm^−1^ was assigned to O–H stretching vibration, and the band at 2935.91 cm^−1^ was attributed to C–H stretching vibration. The absorption bands at 1654.68 and 1404.85 cm^−1^ were associated with carboxyl-related vibrations, consistent with the presence of uronic acid. The absorption at 1249.87 cm^−1^ was assigned to S=O stretching vibration of sulfate groups, whereas the band around 811.67 cm^−1^ supported the presence of sulfate ester-related vibrations. In addition, the absorption around 1048.71 cm^−1^ was consistent with C–O–C and C–O stretching vibrations in the glycosidic structure. These FT-IR features were in accordance with the chemical composition results, supporting the finding that P0.7 was an acidic sulfated polysaccharide.

**Figure 2:**
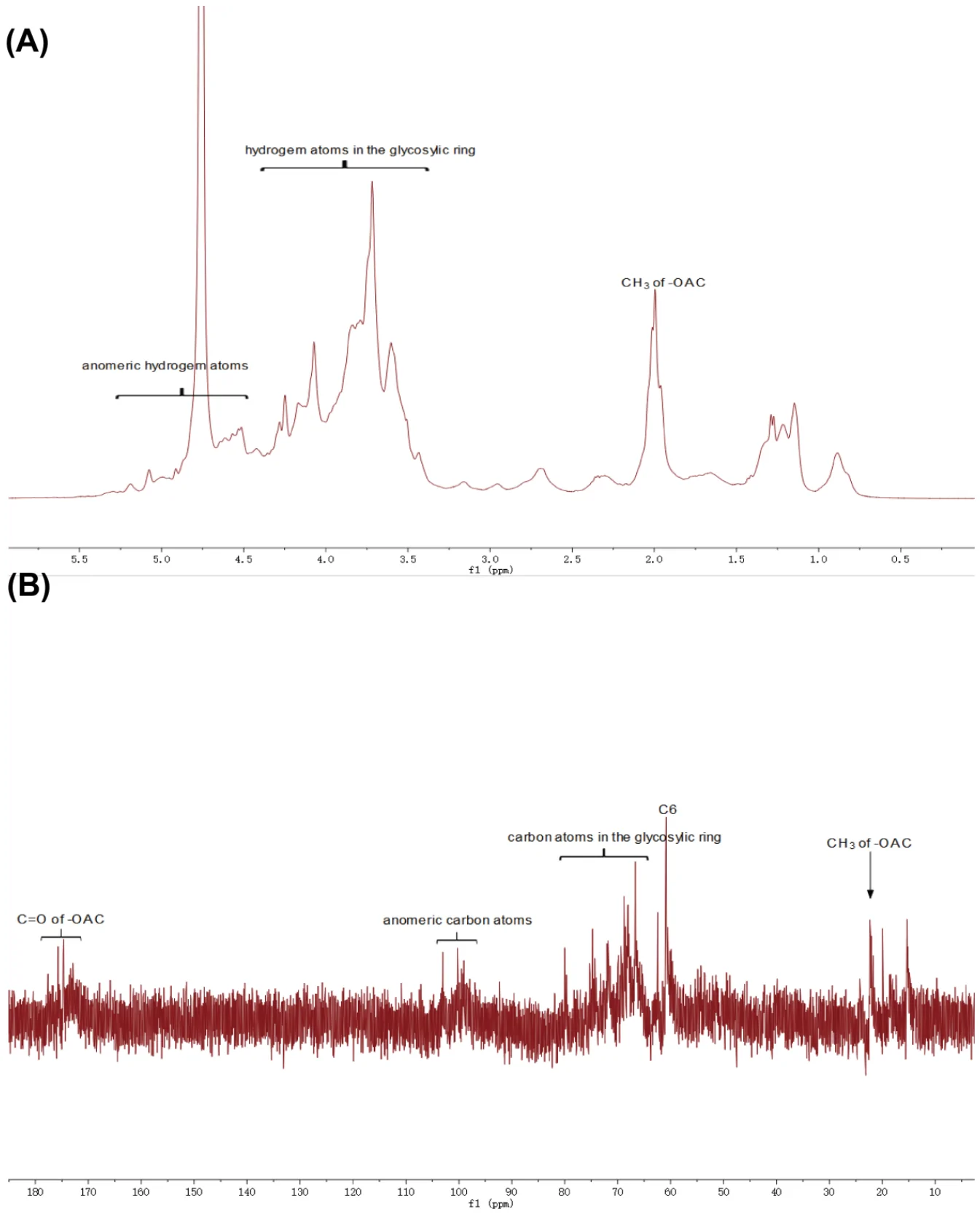
NMR characterization of P0.7. (**A**) ^1^H NMR spectrum of P0.7. (**B**) ^13^C NMR spectrum of P0.7.

NMR spectroscopy provided further information on the structural features of P0.7. In the ^1^H NMR spectrum, signals in the δ 4.5–5.3 ppm region were assigned to anomeric protons, whereas signals distributed between δ 3.2 and 4.2 ppm corresponded to protons in the glycosidic ring (Figure 2A). The signal around δ 2.0 ppm indicated the presence of methyl protons from possible O-acetyl groups. In the ^13^C NMR spectrum, signals around δ 95–105 ppm were assigned to anomeric carbons, whereas signals in the δ 60–80 ppm region corresponded to glycosidic ring carbons (Figure 2B). Signals around δ 170–175 ppm and δ 20–22 ppm were consistent with the carbonyl and methyl carbons of O-acetyl groups, respectively. These NMR characteristics further indicated that P0.7 possessed a complex heteropolysaccharide structure with possible O-acetyl substitution.

Collectively, the chromatographic, compositional, molecular weight, FT-IR, NMR, and SEM analyses demonstrated that P0.7 was a relatively homogeneous sulfated acidic heteropolysaccharide-rich fraction isolated from sea cucumber cooking liquid. Its low molecular weight, high carbohydrate content, measurable sulfate and uronic acid contents, complex monosaccharide composition, and characteristic polysaccharide spectroscopic signals provided the structural basis for subsequent evaluation of its antitumor activity.

### 2.2. In Vitro Evaluation of P0.7

#### 2.2.1. Cell Growth, Apoptosis, and Cell Cycle Distribution

**Figure 3:**
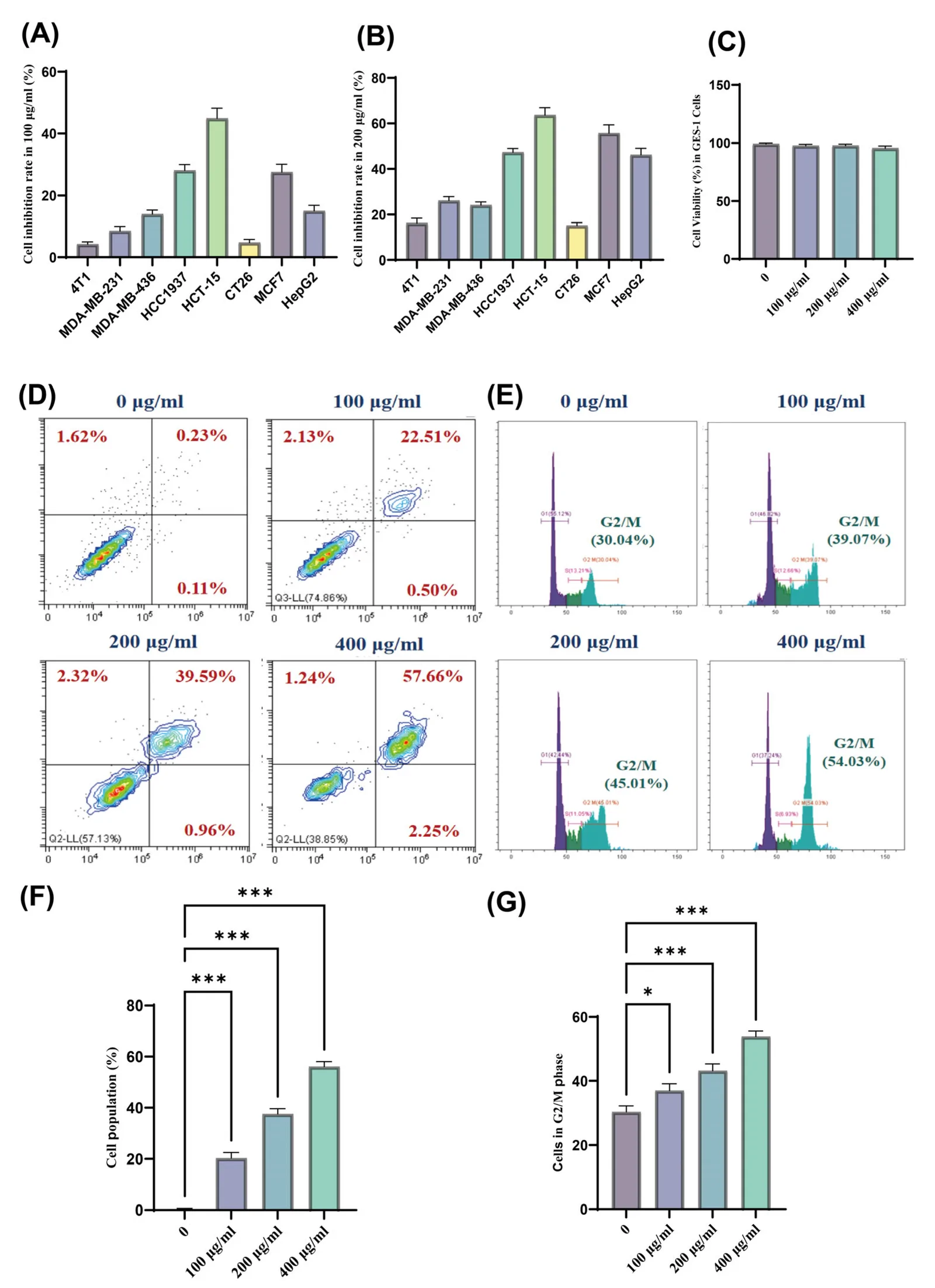
P0.7 inhibits HCT-15 cell growth and induces apoptosis and G2/M-phase accumulation. (**A**,**B**) Inhibitory effects of P0.7 on different tumor cell lines at 100 μg/mL (**A**) and 200 μg/mL (**B**). (**C**) Cell viability (%) in GES-1 cells. (**D**) Representative flow cytometric plots showing apoptotic HCT-15 cells after treatment with P0.7 at 0, 100, 200, and 400 μg/mL for 48 h. (**E**) Representative cell cycle distribution profiles of HCT-15 cells after P0.7 treatment. (**F**) Quantitative analysis of apoptotic cell populations. (**G**) Quantitative analysis of cells in the G2/M phase. Data are presented as mean ± SD (*n* = 3). Statistical significance was determined by one-way ANOVA followed by Tukey’s post hoc test. * *p* < 0.05, *** *p* < 0.001 versus control.

The inhibitory effect of P0.7 was first evaluated in different tumor cell lines to identify the cell type most responsive to this fraction. At 100 μg/mL, P0.7 exerted relatively weak inhibition on 4T1, MDA-MB-231, MDA-MB-436, CT26, and HepG2 cells, with inhibition rates of approximately 4%, 8%, 14%, 5%, and 15%, respectively (Figure 3A). Moderate inhibition was observed in HCC1937 and MCF7 cells, with inhibition rates of approximately 28% and 27%, respectively. Among the tested cell lines, HCT-15 cells showed the highest sensitivity to P0.7, with an inhibition rate of approximately 45% at 100 μg/mL. When the concentration was increased to 200 μg/mL, the inhibitory effect was further enhanced. HCT-15 cells remained the most responsive cell line, with an inhibition rate of approximately 63%, followed by MCF7, HCC1937, and HepG2 cells, with inhibition rates of approximately 56%, 47%, and 46%, respectively (Figure 3B). Meanwhile, as shown in Figure 3C, P0.7 did not exhibit obvious cytotoxicity toward the normal human gastric epithelial cell line GES-1. Therefore, HCT-15 cells, which showed greater sensitivity to P0.7, were selected for subsequent pharmacological evaluation.

Flow cytometry was then used to determine whether the growth inhibition induced by P0.7 was associated with apoptosis. In untreated HCT-15 cells, the proportions of early and late apoptotic cells were 0.11% and 0.23%, respectively (Figure 3D). After treatment with 100, 200, and 400 μg/mL of P0.7, the late apoptotic population increased to 22.51%, 39.59%, and 57.66%, respectively, whereas the early apoptotic population increased to 0.50%, 0.96%, and 2.25%, respectively. Accordingly, the total apoptotic population increased from 0.34% in untreated cells to approximately 23.01%, 40.55%, and 59.91% after treatment with 100, 200, and 400 μg/mL of P0.7, respectively (Figure 3F). These results demonstrated that P0.7 markedly promoted apoptosis in HCT-15 cells.

Cell cycle analysis further showed that P0.7 altered cell cycle distribution in HCT-15 cells. The proportion of cells in the G2/M phase increased from 30.04% in untreated cells to 39.07%, 45.01%, and 54.03% after treatment with 100, 200, and 400 μg/mL of P0.7, respectively (Figure 3E,G). This increase was accompanied by a corresponding redistribution of cells in other phases, indicating that P0.7-induced G2/M-phase accumulation. These findings indicate that P0.7 inhibited HCT-15 cell growth by promoting apoptosis and interfering with cell cycle progression.

#### 2.2.2. ROS Accumulation and Mitochondrial Membrane Potential

**Figure 4:**
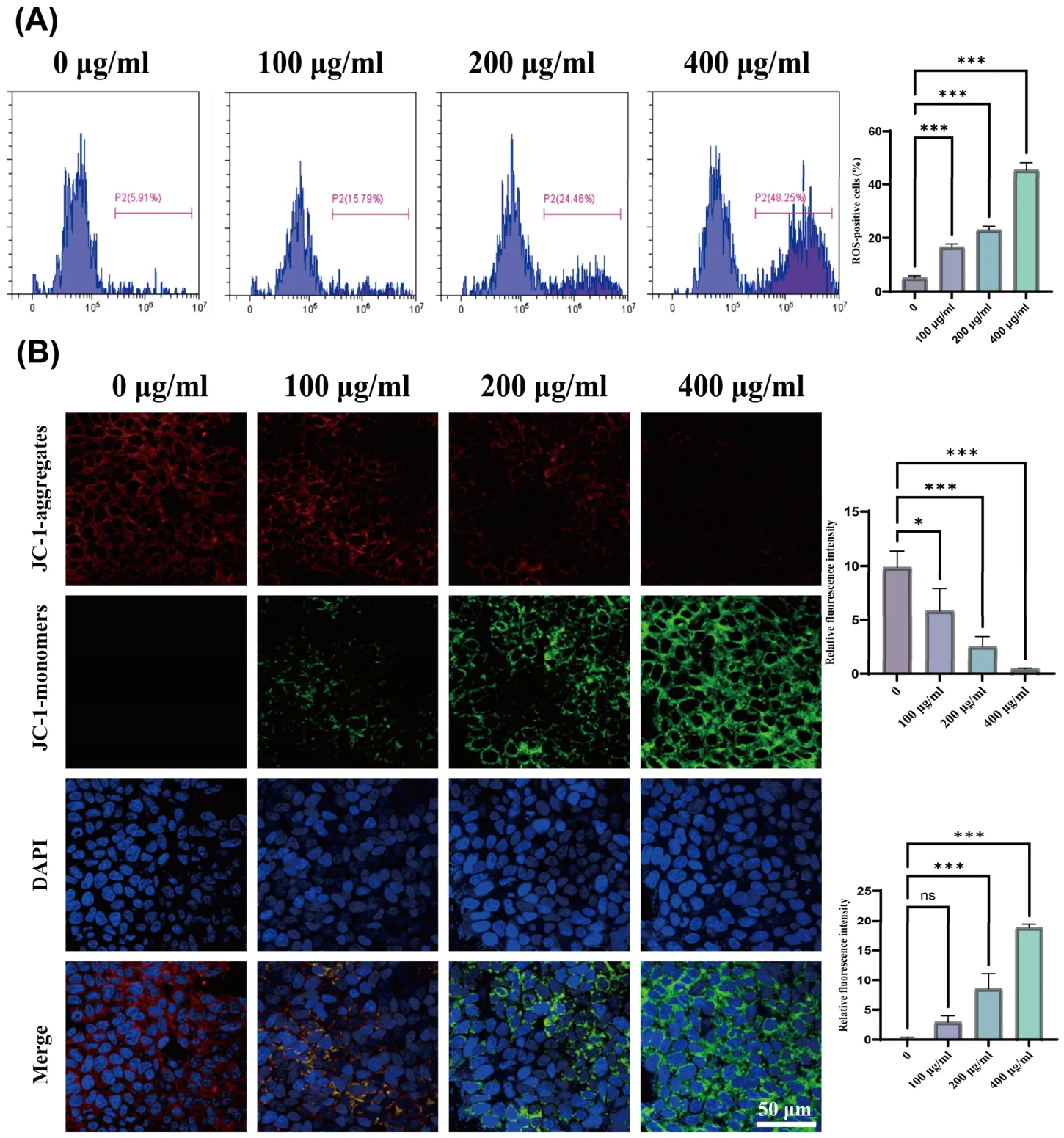
P0.7 increases intracellular ROS accumulation and disrupts mitochondrial membrane potential in HCT-15 cells. (**A**) Representative flow cytometric histograms and quantitative analysis of intracellular ROS levels in HCT-15 cells treated with P0.7 at 0, 100, 200, and 400 μg/mL for 48 h. P2: ROS-positive cell population. (**B**) Representative JC-1 staining images showing mitochondrial membrane potential changes after P0.7 treatment. Red fluorescence indicates JC-1 aggregates, whereas green fluorescence indicates JC-1 monomers. DAPI was used for nuclear staining. Quantitative analysis of red and green fluorescence intensities is shown on the right. Scale bar = 50 μm. Data are presented as mean ± SD (*n* = 3). Statistical significance was determined by one-way ANOVA followed by Tukey’s post hoc test. * *p* < 0.05, *** *p* < 0.001 versus control. ns: no significance.

To further explore the cellular events associated with P0.7-induced apoptosis, intracellular ROS levels and mitochondrial membrane potential were examined. Flow cytometric analysis showed that the ROS-positive population increased from 5.91% in untreated cells to 15.79%, 24.46%, and 48.25% after treatment with 100, 200, and 400 μg/mL of P0.7, respectively (Figure 4A). This result demonstrated that P0.7 markedly increased intracellular ROS accumulation in HCT-15 cells.

Mitochondrial membrane potential was subsequently evaluated by JC-1 staining. Untreated cells showed strong red JC-1 aggregate fluorescence and weak green JC-1 monomer fluorescence, indicating relatively preserved mitochondrial membrane potential (Figure 4B). After P0.7 treatment, the red fluorescence signal gradually decreased, whereas the green fluorescence signal increased. Quantitative analysis showed that the relative red fluorescence intensity decreased from approximately 9 in untreated cells to approximately 6, 2, and less than 1 after treatment with 100, 200, and 400 μg/mL of P0.7, respectively. Conversely, the relative green fluorescence intensity increased from approximately 1 in untreated cells to approximately 3, 8, and 19 at the corresponding concentrations. These results indicated that P0.7 induced the dissipation of mitochondrial membrane potential in HCT-15 cells, suggesting subsequent mitochondrial dysfunction.

#### 2.2.3. Apoptotic Proteins and DNA Damage Response

**Figure 5:**
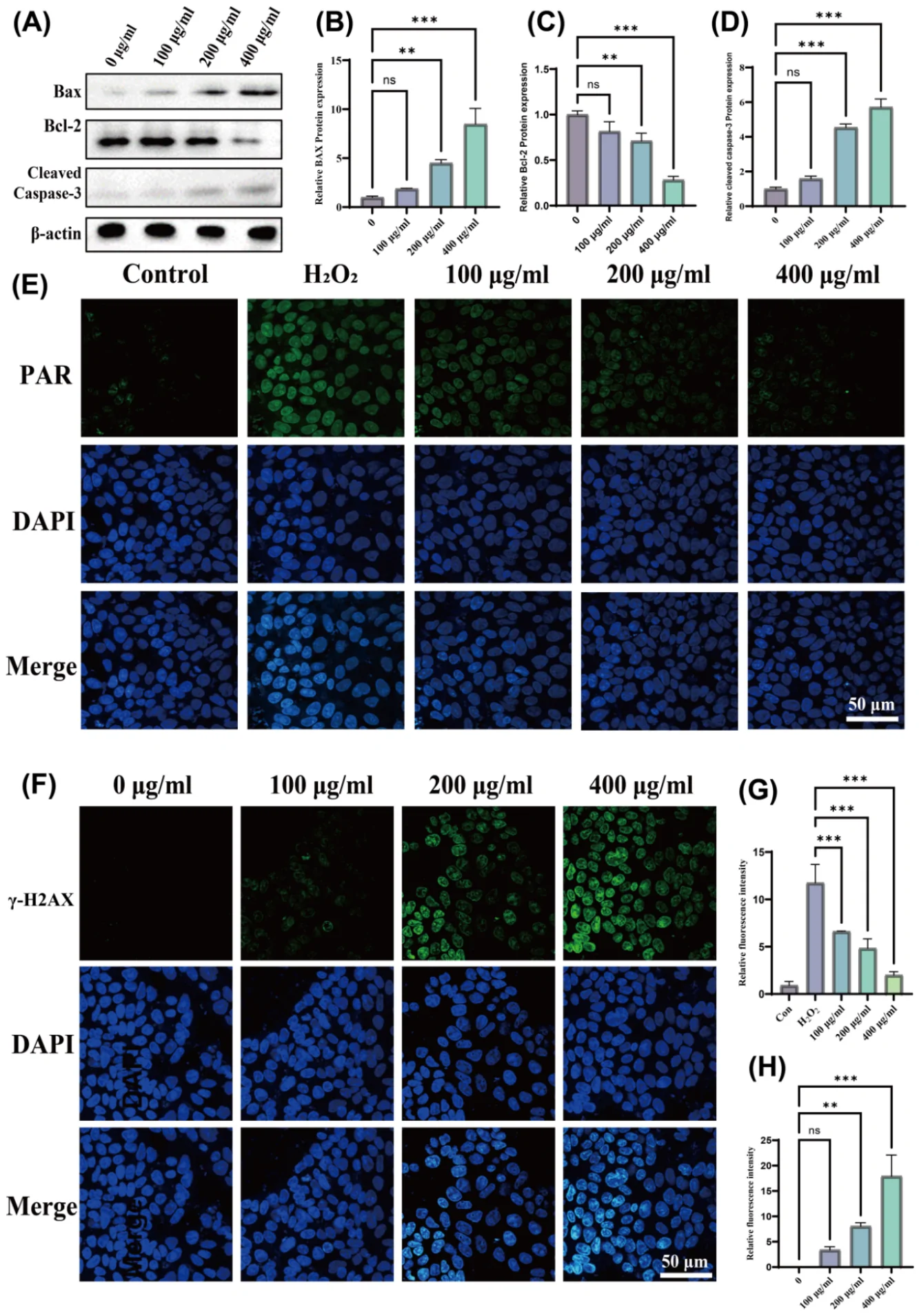
P0.7 regulates apoptosis-related proteins and DNA damage-response markers in HCT-15 cells. (**A**) Representative Western blot bands of Bax, Bcl-2, cleaved caspase-3, and β-actin in HCT-15 cells treated with P0.7 at 0, 100, 200, and 400 μg/mL for 48 h. (**B**–**D**) Quantitative analysis of Bax, Bcl-2, and cleaved caspase-3 expression. (**E**) Representative immunofluorescence images of PAR staining. H_2_O_2_ was used as a positive control for oxidative DNA damage. (**F**) Representative immunofluorescence images of γ-H2AX staining after P0.7 treatment for 48 h. (**G**,**H**) Quantitative analysis of PAR and γ-H2AX fluorescence intensities. DAPI was used for nuclear staining. Scale bars = 50 μm. Data are presented as mean ± SD (*n* = 3). Statistical significance was determined by one-way ANOVA followed by Tukey’s post hoc test. ** *p* < 0.01, *** *p* < 0.001 versus control. ns: no significance.

Western blotting was performed to further assess the involvement of apoptosis-related signaling. P0.7 increased the expression of the proapoptotic protein Bax and decreased the expression of the antiapoptotic protein Bcl-2 in HCT-15 cells (Figure 5A–C). Compared with untreated cells, Bax expression increased to approximately 1.8-, 4.3-, and 8.5-fold after treatment with 100, 200, and 400 μg/mL of P0.7, respectively. In contrast, Bcl-2 expression decreased to approximately 0.8-, 0.7-, and 0.3-fold of the untreated level at the corresponding concentrations. Cleaved caspase-3 was also increased, especially after treatment with 200 and 400 μg/mL of P0.7 (Figure 5D). These results were consistent with the apoptosis and JC-1 staining data and supported the involvement of mitochondria-associated apoptotic signaling in P0.7-treated HCT-15 cells.

The DNA damage response was further evaluated by detecting PAR and γ-H2AX signals. H_2_O_2_ markedly increased PAR fluorescence and served as a positive control for oxidative DNA damage (Figure 5E,G). Compared with the H_2_O_2_ group, P0.7-treated cells showed lower PAR fluorescence, and the PAR signal decreased from approximately 7 at 100 μg/mL to approximately 4 and 2 at 200 and 400 μg/mL, respectively. This reduction indicated that PAR formation was decreased after P0.7 treatment, suggesting that PARP-related DNA repair responses may be impaired under P0.7-induced cellular stress.

In contrast, γ-H2AX immunofluorescence showed a clear increase after P0.7 exposure. The relative γ-H2AX fluorescence intensity increased from the basal level in untreated cells to approximately 3-, 8-, and 18-fold after treatment with 100, 200, and 400 μg/mL of P0.7, respectively (Figure 5F,H). Since γ-H2AX is closely associated with DNA damage-response signaling, these results indicated that P0.7 activated γ-H2AX-related DNA damage-response signaling in HCT-15 cells. These cellular events were associated with the inhibitory effect of P0.7 on HCT-15 cell growth.

### 2.3. In Vivo Evaluation of P0.7

#### 2.3.1. Tumor Growth and Body Weight Changes

**Figure 6:**
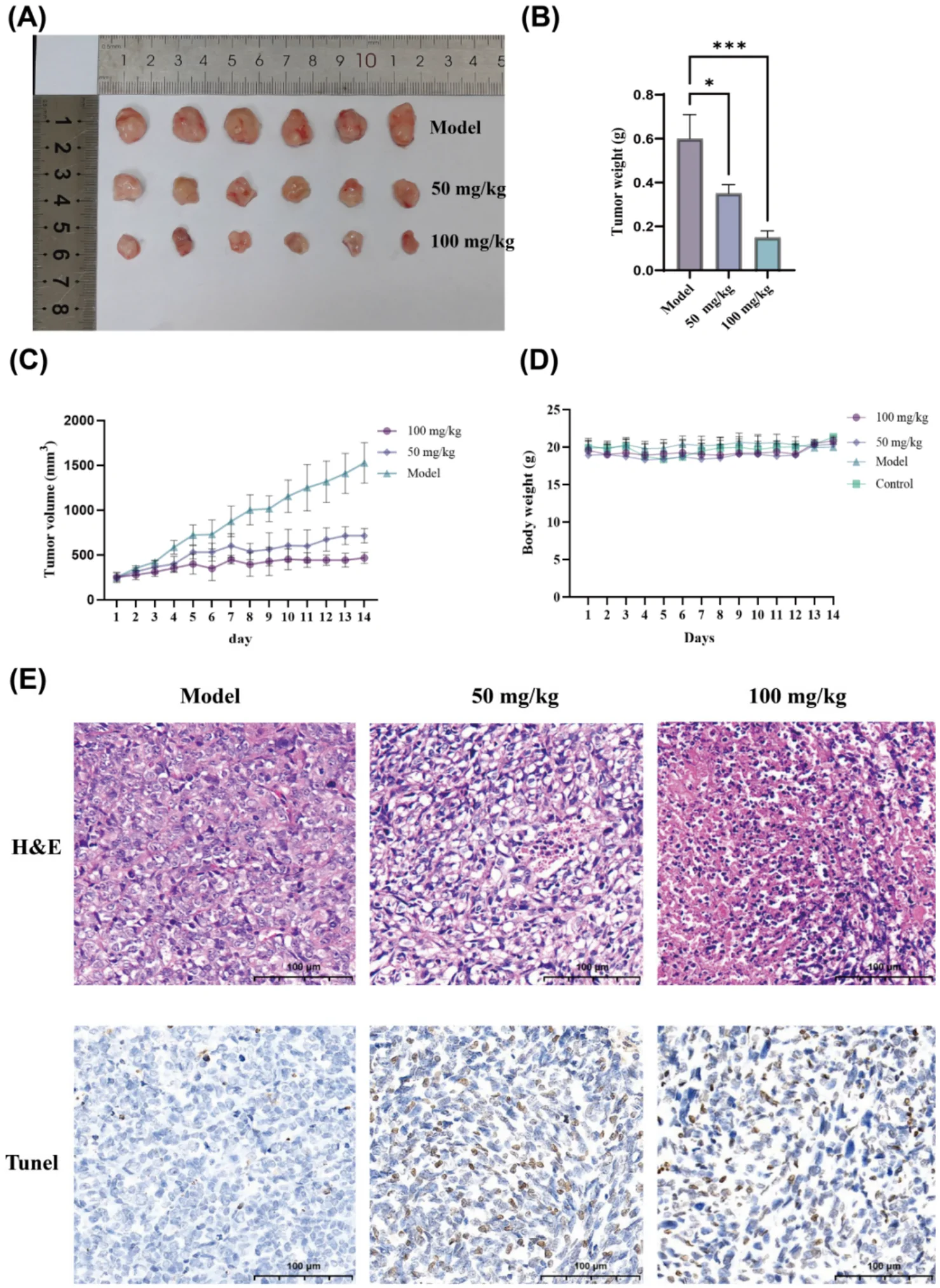
P0.7 suppresses tumor growth in an HCT-15 xenograft mouse model within 14 days. (**A**) Representative images of excised tumors from the model group and P0.7-treated groups. (**B**) Terminal tumor weight. (**C**) Tumor volume changes during the observation period. (**D**) Body weight changes during P0.7 treatment. (**E**) Representative H&E and TUNEL staining images of tumor sections. Scale bars = 100 μm. Tumor volume, tumor weight and body weight data are presented as mean ± SD (*n* = 6). * *p* < 0.05, *** *p* < 0.001 versus control.

To evaluate the antitumor activity of P0.7 in vivo, an HCT-15 xenograft mouse model was established, and tumor-bearing mice were treated with P0.7 at 50 or 100 mg/kg. At the beginning of treatment, the mean tumor volumes were comparable among the model, 50 mg/kg, and 100 mg/kg groups, indicating similar baseline tumor burdens. During the observation period, tumor volume increased progressively in the model group, whereas P0.7 treatment markedly slowed tumor growth (Figure 6C). On day 14, the mean tumor volume reached 1530.25 ± 228.60 mm^3^ in the model group, but was reduced to 714.01 ± 80.07 mm^3^ and 467.72 ± 61.62 mm^3^ in the 50 and 100 mg/kg P0.7 groups, respectively. The corresponding inhibition rates based on terminal tumor volume were approximately 53.3% and 69.4%, respectively.

Consistent with the tumor volume data, excised tumors from P0.7-treated mice were visibly smaller than those from the model group (Figure 6A). Quantitative analysis of terminal tumor weight further confirmed the inhibitory effect of P0.7. The mean tumor weight was 0.60 ± 0.11 g in the model group, whereas it decreased to 0.35 ± 0.04 g and 0.15 ± 0.03 g after treatment with 50 and 100 mg/kg of P0.7, respectively (Figure 6B). These values corresponded to tumor weight inhibition rates of approximately 41.7% and 75.0%, respectively. In addition, no obvious body weight loss was observed during P0.7 treatment (Figure 6D), suggesting that P0.7 administration was not associated with apparent systemic burden under the present experimental conditions.

#### 2.3.2. Histological and Immunohistochemical Analysis

Histological examination was performed to further assess the effect of P0.7 on HCT-15 xenograft tumors. H&E staining showed that tumors from the model group exhibited dense cellularity and compact tumor architecture. In contrast, P0.7-treated tumor tissues displayed reduced tumor cell density and increased degenerative areas, particularly in the 100 mg/kg group (Figure 6E). TUNEL staining showed limited TUNEL-positive cells in the model group, whereas P0.7 treatment increased TUNEL-positive signals in tumor sections, indicating enhanced apoptosis-associated DNA fragmentation in xenograft tumor tissues (Figure 6E).

**Figure 7:**
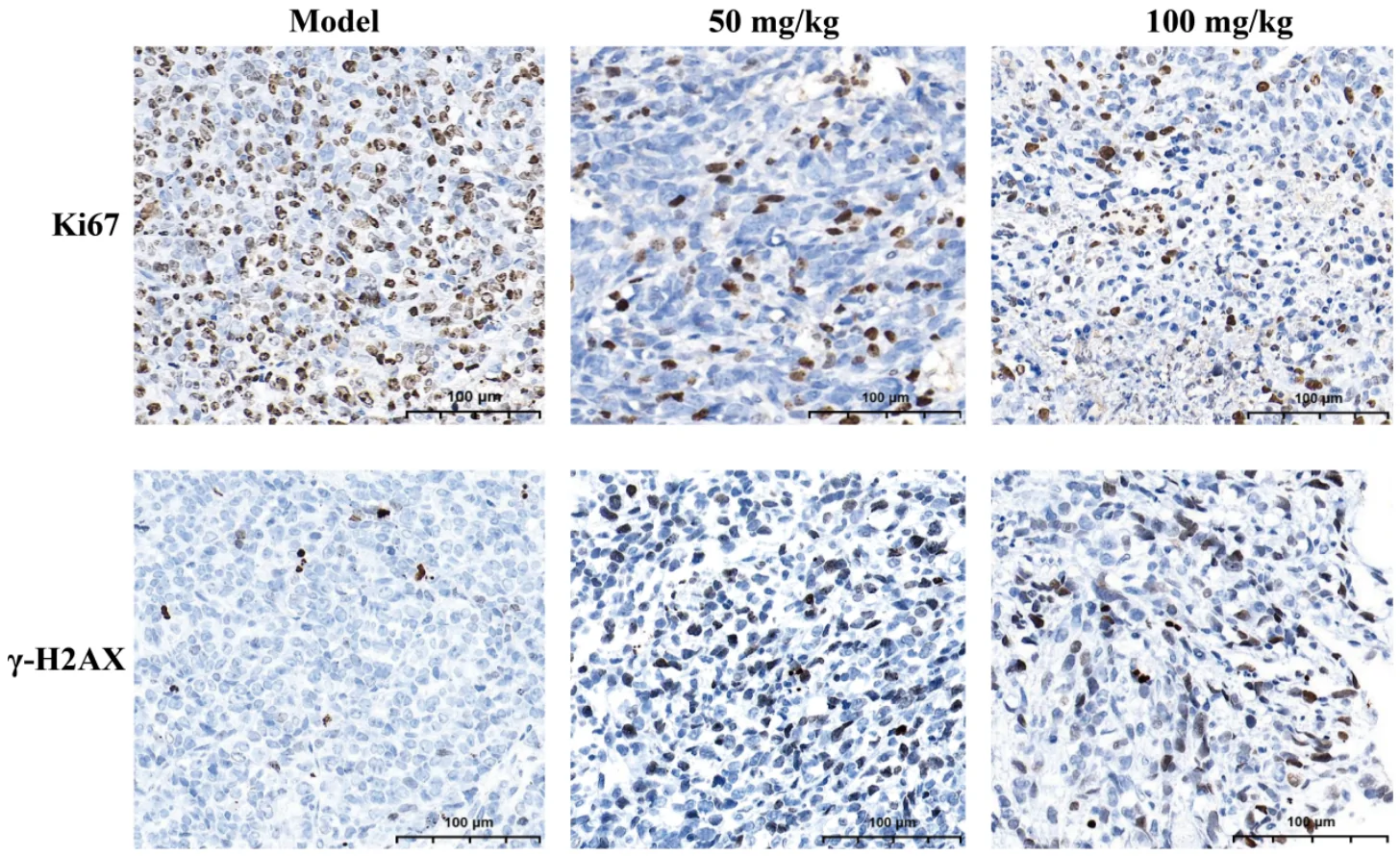
P0.7 modulates Ki67 and γ-H2AX staining in HCT-15 xenograft tumor tissues. Representative immunohistochemical staining images of Ki67 and γ-H2AX in tumor sections from the model group and P0.7-treated groups. Ki67 was used as a marker of tumor cell proliferation, whereas γ-H2AX was assessed as a marker associated with the DNA damage response. Scale bars = 100 μm.

Immunohistochemical staining was further performed to evaluate tumor cell proliferation and DNA damage-response-related changes. Ki67-positive nuclear staining was abundant in the model group, indicating active proliferative status in HCT-15 xenograft tumors. After P0.7 treatment, Ki67-positive signals were reduced, suggesting decreased proliferative activity in tumor tissues (Figure 7). In parallel, γ-H2AX-positive nuclear signals were increased in P0.7-treated tumors compared with the model group, which was consistent with activation of DNA damage-response-related signaling (Figure 7). Collectively, these in vivo results demonstrated that P0.7 suppressed HCT-15 xenograft tumor growth, accompanied by reduced tumor cell proliferation, increased apoptosis-associated DNA fragmentation, and enhanced γ-H2AX-related DNA damage response.

## 3. Discussion

In the present study, P0.7 was isolated from sea cucumber cooking liquid by anion-exchange chromatography and characterized as a low-molecular-weight sulfated acidic heteropolysaccharide-rich fraction. The predominant RI chromatographic peak, together with the estimated molecular weight of approximately 6.7 kDa, indicated the effective enrichment of P0.7 from the complex cooking liquid matrix. Its high total sugar content (83.61 ± 3.65%), together with measurable sulfate (13.67 ± 2.48%) and uronic acid (9.98 ± 1.22%) contents, supported its assignment as an acidic sulfated polysaccharide-rich fraction. FT-IR analysis revealed typical polysaccharide absorptions, sulfate ester-related vibrations, and carboxyl-associated signals, while one-dimensional NMR spectra showed anomeric- and glycosidic-ring resonances, together with signals tentatively assigned to O-acetyl substitution. These physicochemical characteristics provide a structural basis for the subsequent biological evaluation, as molecular size, monosaccharide composition, acidic residues, sulfate substitution, and chain organization are closely related to the biological properties of marine polysaccharides [10].

The monosaccharide composition of P0.7 was related to, but distinct from, previously reported polysaccharide fractions from sea cucumber cooking liquid. Similar to reported cooking liquid-derived polysaccharides, P0.7 contained galactose, mannose, glucose, amino sugars, uronic acid residues, and fucose-related components [18,19,20,21]. However, its relative monosaccharide distribution was distinctive: galactose, mannose, and glucose were the predominant residues and collectively accounted for 79.04% of the total monosaccharide composition, whereas fucose represented only 2.96%. Thus, P0.7 should not be regarded as a completely unrelated material compared with reported sea cucumber cooking liquid polysaccharides, but rather as a newly purified fraction with a clearly different compositional pattern. This feature distinguishes P0.7 from previously reported fucose-rich fucoidan from sea cucumber cooking liquid, in which fucose was the dominant monosaccharide and the sulfate content was higher [21].

P0.7 was recovered from the fraction eluted with 0.7 mol/L NaCl during anion-exchange chromatography. Under the applied separation conditions, this relatively low salt eluate indicates weaker anionic retention on the positively charged column matrix, which is consistent with the comparatively modest sulfate content of P0.7. In sea cucumber sulfated fucans, sulfate substitution is generally associated with fucosyl residues; therefore, the low fucose abundance of P0.7 may partly explain its relatively low sulfate level [15]. In this context, the 0.7 mol/L NaCl elution, moderate sulfate content, and low fucose proportion are mutually consistent rather than independent observations. Together with its dominant galactose, mannose, and glucose composition, these features support the identification of P0.7 as a newly purified and compositionally distinctive sulfated acidic heteropolysaccharide-rich fraction from sea cucumber cooking liquid.

The isolation of P0.7 expands the value of sea cucumber cooking liquid beyond the recovery of crude processing byproducts. Previous studies have shown that polysaccharides can be recovered from sea cucumber processing waste liquid and that cooking liquid-derived polysaccharide fractions exhibit antioxidant, metabolic regulatory, gut microbiota modulating, anti-*Helicobacter pylori*, and oxidative injury protective activities [18,19,20,21,22,23]. Compared with these studies, the present work links chromatographic purification and physicochemical characterization of a defined polysaccharide-rich fraction with antitumor activity in both cellular and xenograft models. Therefore, P0.7 provides further evidence that sea cucumber cooking liquid can be transformed from an underutilized processing stream into a source of purified bioactive marine polysaccharide fractions with potential for high-value functional development.

Among the tumor cell lines tested, HCT-15 colorectal cancer cells showed the greatest sensitivity to P0.7, with inhibition rates of approximately 45% and 63% at 100 and 200 μg/mL, respectively. In HCT-15 cells, P0.7 increased the apoptotic population and induced G2/M-phase accumulation, indicating that its growth-inhibitory effect was associated with both enhanced cell death and altered cell cycle progression. The increase in G2/M-phase cells was accompanied by elevated γ-H2AX signaling, suggesting that cell cycle disturbance occurred together with DNA damage-response-associated signaling.

P0.7 treatment also caused concentration-dependent intracellular ROS accumulation and a marked loss of mitochondrial membrane potential (ΔΨm). These changes were accompanied by increased Bax expression, decreased Bcl-2 expression, and elevated cleaved caspase-3 levels. The coordinated regulation of ROS production, ΔΨm, Bax/Bcl-2 balance, and caspase-3 cleavage supports the involvement of mitochondria-associated apoptosis in P0.7-treated HCT-15 cells. Excessive ROS accumulation can disrupt mitochondrial homeostasis and activate apoptosis-related signaling in cancer cells [24]. Therefore, oxidative stress-associated mitochondrial dysfunction may contribute to the cytotoxic response induced by P0.7.

P0.7 exposure was further associated with changes in DNA damage-response-related signaling. γ-H2AX fluorescence increased after P0.7 treatment in HCT-15 cells, and enhanced γ-H2AX staining was also observed in xenograft tumor tissues, indicating activation of γ-H2AX-related DNA damage-response signaling [25,26]. In the PAR assay, H_2_O_2_ challenge markedly increased PAR-associated fluorescence, whereas P0.7 pretreatment reduced the PAR signal relative to the H_2_O_2_ group. As PAR is the principal product of PARP activation, this result suggests that P0.7 altered PARylation-associated signaling following H_2_O_2_-induced oxidative DNA damage [27]. The concurrent occurrence of ROS accumulation, ΔΨm loss, G2/M-phase accumulation, apoptosis-associated protein regulation, altered PAR signaling, and increased γ-H2AX expression suggests that oxidative stress, mitochondrial dysfunction, and DNA damage-response-associated signaling may collectively participate in the suppression of HCT-15 cell growth. However, the present results do not establish a strict causal sequence among these events.

The in vivo results further supported the biological relevance of P0.7. In the HCT-15 xenograft model, P0.7 reduced terminal tumor volume and tumor weight relative to the model group. This tumor growth suppressive effect was accompanied by reduced Ki67 staining, increased TUNEL-positive signals, and enhanced γ-H2AX staining in tumor tissues. These histological and immunohistochemical changes were consistent with the in vitro findings, indicating that P0.7-mediated tumor suppression was accompanied by reduced proliferative activity, enhanced apoptosis-associated DNA fragmentation, and increased DNA damage-response-related signaling. Body weights remained stable throughout the treatment period, with no obvious treatment-related weight loss. Although complete tumor regression was not observed, P0.7 markedly attenuated tumor growth under the present experimental conditions.

In conclusion, P0.7 is a newly purified, compositionally distinct, low-molecular-weight sulfated acidic heteropolysaccharide-rich fraction isolated from sea cucumber cooking liquid. It is characterized by the predominance of galactose, mannose, and glucose, together with low fucose abundance, moderate sulfate content, and elution at 0.7 mol/L NaCl. P0.7 suppressed HCT-15 colorectal cancer cell growth in vitro and inhibited HCT-15 xenograft tumor growth in vivo. These effects were associated with ROS accumulation, mitochondrial membrane potential loss, G2/M-phase accumulation, regulation of apoptosis-associated proteins, altered PAR-associated signaling, and activation of γ-H2AX-related DNA damage-response signaling. These findings provide additional experimental evidence supporting further pharmaceutical investigation of bioactive polysaccharides derived from sea cucumber processing resources.

A notable feature of P0.7 is that its monosaccharide composition differs from that of previously reported fucose-rich polysaccharides from sea cucumber cooking liquid. Its relatively low fucose content may be associated with its moderate sulfate content and relatively weak anionic retention, as reflected by elution at 0.7 mol/L NaCl during anion-exchange chromatography. Nevertheless, several limitations should be acknowledged. The detailed glycosidic linkages, branching patterns, sulfate substitution sites, and higher-order structure of P0.7 remain unresolved. The causal role of ROS in P0.7-induced apoptosis was not directly verified using ROS-scavenger rescue experiments, and the biological evaluation was mainly limited to HCT-15 cells and a single immunodeficient xenograft model. In addition, evaluation in normal cells was limited, and comprehensive pharmacokinetic and systemic toxicity studies were not performed. Further structural characterization, mechanistic validation, evaluation in additional tumor and normal cell models, and systematic pharmacokinetic and safety studies are therefore required to clarify the structure–activity relationships and translational potential of P0.7.

## 4. Materials and Methods

### 4.1. Materials and Reagents

Sea cucumber cooking liquid generated during thermal processing was supplied by Homey Group Co., Ltd. (Rongcheng City, China) and used as the starting material for crude polysaccharide preparation. Papain, trypsin, and monosaccharide standards, including L-fucose, D-galactosamine, L-rhamnose, D-arabinose, D-glucosamine, D-galactose, D-glucose, D-xylose, D-mannose, D-fructose, D-ribose, D-galacturonic acid, L-guluronic acid, D-glucuronic acid, and D-mannuronic acid, were purchased from Shanghai Yuanye Bio-Technology Co., Ltd. (Shanghai, China). Deuterium oxide (D_2_O) was purchased from Sigma-Aldrich (St. Louis, MO, USA). Cetylpyridinium chloride, trifluoroacetic acid, sodium hydroxide, sodium acetate, sodium nitrate, sodium azide, ethanol, and other analytical-grade reagents were obtained from commercial suppliers.

MTT, Annexin V-FITC/propidium iodide (PI) apoptosis detection reagents, RNase A, PI, DCFH-DA, JC-1 staining solution, and DAPI were purchased from Beyotime Institute of Biotechnology (Shanghai, China). TUNEL staining reagents and secondary antibodies were obtained from commercial suppliers. Primary antibodies against Bax, Bcl-2, cleaved caspase-3, Ki67, and β-actin were purchased from Proteintech Group, Inc. (Wuhan, China). The anti-γ-H2AX antibody was obtained from Beyotime (C2035), whereas the anti-PAR antibody was purchased from Enzo Life Sciences (ELSB-LSA-216-0100, Farmingdale, NY, USA). All antibodies were used according to the manufacturers’ instructions.

### 4.2. Preparation of Crude Polysaccharide and Purification of P0.7

Sea cucumber cooking liquid was concentrated and lyophilized. Lyophilized cooking liquid powder (100.00 g) was suspended in 3.0 L citrate buffer (pH 6.0). To reduce protein-associated components, the suspension was subjected to sequential enzymatic treatment with papain and trypsin. The enzymatic reaction was terminated by heating at 100 °C. After cooling to room temperature, the mixture was centrifuged at 8000 rpm for 15 min. A 10% CPC solution was added to the supernatant, and the mixture was kept at room temperature for 24 h. The resulting precipitate was collected by centrifugation at 8000 rpm for 15 min, dissolved in 1.50 L of NaCl–ethanol solution, and reprecipitated by addition of 3.0 L of 95% ethanol at 4 °C for 24 h. The precipitate was recovered by centrifugation at 8000 rpm for 15 min, washed two to three times with anhydrous ethanol, and dialyzed against deionized water at 4 °C for 72 h using dialysis tubing with a molecular weight cutoff of 6000–8000 Da. The dialysis water was replaced every 12 h. The retained solution was frozen at −80 °C for 12 h and lyophilized for 24 h to obtain the crude polysaccharide fraction.

The crude polysaccharide fraction was dissolved in distilled water and loaded onto a Q-Sepharose Fast Flow anion-exchange column. The column was eluted with a NaCl gradient, and carbohydrate-containing fractions were monitored using the phenol sulfuric acid method at 490 nm [28]. The fraction eluted at 0.7 mol/L NaCl was pooled and designated P0.7. The pooled fraction was dialyzed against deionized water, concentrated, and lyophilized for subsequent physicochemical characterization and biological evaluation. The yield of P0.7 was calculated relative to the crude polysaccharide fraction as follows: yield (%) = mass of lyophilized P0.7/mass of crude polysaccharide fraction × 100.

### 4.3. Chemical Composition Analysis

The total carbohydrate content of P0.7 was determined by the phenol–sulfuric acid method using glucose as the standard [28]. Protein content was measured by the Coomassie brilliant blue method using bovine serum albumin as the standard [29]. Uronic acid content was determined by the sulfuric acid–carbazole method using galacturonic acid as the standard [30]. Sulfate content was measured by the gelatin–barium chloride turbidimetric method using potassium sulfate as the standard [31]. All measurements were performed in triplicate and expressed as percentages of the dry sample weight.

### 4.4. Molecular Weight Determination

The molecular weight of P0.7 was evaluated by high-performance gel permeation chromatography coupled with refractive-index detection (HPGPC-RI). P0.7 was dissolved in 1 mL of mobile phase, ultrasonicated, centrifuged, and filtered through a 0.22 μm membrane before analysis. Chromatography was performed using a Thermo U3000 liquid chromatography system coupled with an Optilab T-rEX refractive-index detector (Wyatt Technology, Santa Barbara, CA, USA). OHpak SB-805 HQ and SB-803 HQ columns (300 × 8 mm) were connected in series. The mobile phase consisted of 0.02% NaN_3_ in 0.1 mol/L NaNO_3_. The column temperature was maintained at 45 °C, the flow rate was 0.6 mL/min, the injection volume was 100 μL, and the analysis time was 50 min.

### 4.5. Monosaccharide Composition Analysis

Monosaccharide composition was determined by high-performance anion-exchange chromatography using a Thermo ICS-5000 ion chromatography system [32]. P0.7 was hydrolyzed with 2 mL of 3 mol/L trifluoroacetic acid at 110 °C for 3 h. The hydrolysate was dried under nitrogen, repeatedly washed with methanol, dried again, redissolved in ultrapure water, centrifuged, and filtered through a 0.22 μm membrane. Monosaccharide standards were prepared and analyzed under identical conditions. Separation was performed using a Dionex CarboPac PA20 column (3 × 150 mm, 10 μm). The mobile phases were H_2_O (A), 0.1 mol/L NaOH (B), and 0.1 mol/L NaOH containing 0.2 mol/L sodium acetate (C). The gradient program (A/B/C, *v*/*v*/*v*) was 95/5/0 at 0 min, 85/5/10 at 26 min, 85/5/10 at 42 min, 60/0/40 at 42.1 min, 60/40/0 at 52 min, and 95/5/0 at 52.1 and 55 min. The flow rate was 0.5 mL/min, the column temperature was 30 °C, and the injection volume was 5 μL. The molar percentage of each monosaccharide was calculated from the molar amounts determined using the corresponding calibration curves.

### 4.6. FT-IR, NMR, and SEM Analyses

For FT-IR analysis, 2 mg of P0.7 was mixed with 200 mg of dried KBr, finely ground, and pressed into a transparent disk. Spectra were recorded over the range of 4000–400 cm^−1^. For NMR analysis, 50 mg of P0.7 was exchanged with D_2_O three times and finally dissolved in 0.5 mL D_2_O. One-dimensional ^1^H and ^13^C NMR spectra were acquired at 25 °C using a Bruker AVANCE III HD 600 spectrometer (Billerca, MA, USA) operating at 600 MHz. The surface morphology of lyophilized P0.7 was examined by scanning electron microscopy after sputter coating with gold.

### 4.7. Cell Lines and Cell Culture

The tumor cell lines used for the initial growth-inhibition screen were HCT-15, HCC1937, MDA-MB-231, MDA-MB-436, MCF7, 4T1, CT26, GES-1 and HepG2. The cells were purchased from Procell Life Science & Technology Co., Ltd. (Wuhan, China) or obtained from the American Type Culture Collection (ATCC, Manassas, VA, USA). Cells were maintained in DMEM or RPMI 1640 medium, as appropriate for each cell line, supplemented with 10% fetal bovine serum at 37 °C in a humidified incubator containing 5% CO_2_.

### 4.8. Cell Growth Inhibition Assay

Cells were seeded in 96-well plates at 5 × 10^3^ cells per well and treated with P0.7 at 100, 200 or 400 μg/mL for 48 h. MTT was then added, and cells were incubated for an additional 4 h. The optical density was measured at 570 nm using a microplate reader [33]. The inhibition rate was calculated relative to untreated control cells. HCT-15 cells, which showed the highest sensitivity to P0.7 in the screening assay, were selected for subsequent mechanistic experiments.

### 4.9. Cell Cycle and Apoptosis Analyses

HCT-15 cells were seeded in 6-well plates at 1 × 10^5^ cells per well and incubated for 24 h before treatment with P0.7 at 100, 200, or 400 μg/mL for 48 h. Cells were harvested by trypsinization, collected by centrifugation, and washed with PBS. For cell cycle analysis, cells were fixed in 70% ethanol at 4 °C for at least 30 min, resuspended in staining buffer, and incubated with RNase A and PI at 37 °C in the dark for 30 min. For apoptosis analysis, cells were resuspended in Annexin V-FITC binding buffer and stained with Annexin V-FITC and PI for 30 min at room temperature in the dark. Cell cycle distribution and apoptosis were analyzed by flow cytometry (Beckman Coulter, Luzerne, Singapore) [34].

### 4.10. Intracellular ROS Assay

HCT-15 cells were treated with P0.7 at 100, 200, or 400 μg/mL for 48 h. Cells were harvested by trypsinization, washed with PBS, and incubated with 10 μmol/L DCFH-DA at 37 °C for 30 min in the dark. After two washes with PBS to remove excess probe, intracellular ROS levels were measured by flow cytometry within 1 h [35].

### 4.11. Mitochondrial Membrane Potential Assay

Mitochondrial membrane potential was evaluated by JC-1 staining. After treatment with P0.7 at 100, 200, or 400 μg/mL for 48 h, HCT-15 cells were incubated with JC-1 staining solution in fresh culture medium at 37 °C for 30 min. Cells were washed, counterstained with DAPI for nuclear visualization, and imaged using a Leica SP8 laser confocal microscope. Red fluorescence represented JC-1 aggregates, whereas green fluorescence represented JC-1 monomers [36].

### 4.12. Western Blot Analysis

HCT-15 cells were treated with P0.7 at 100, 200, or 400 μg/mL for 48 h. Total proteins were extracted using RIPA lysis buffer, and protein concentrations were determined using a protein quantification assay. Equal amounts of denatured protein were separated by 10% SDS-PAGE and transferred to PVDF membranes. Membranes were blocked with 5% blocking solution for 2 h and incubated overnight at 4 °C with primary antibodies against Bax, Bcl-2, cleaved caspase-3, and β-actin. After three washes with TBST, membranes were incubated with the appropriate secondary antibodies for 2 h at room temperature. Protein signals were visualized using an enhanced chemiluminescence detection system and quantified with ImageJ software (v1.54t). β-actin was used as the loading control.

### 4.13. PAR and γ-H2AX Immunofluorescence Assays

For PAR immunofluorescence analysis, HCT-15 cells were seeded in 20 mm glass-bottom confocal dishes at a density of 5 × 10^4^ cells per dish and allowed to attach for 24 h. Cells were pretreated with P0.7 at 100, 200, or 400 μg/mL for 48 h and subsequently exposed to 600 μM H_2_O_2_ in 1 mL of serum-free medium for 30 min to induce oxidative DNA damage. Untreated cells and cells exposed to H_2_O_2_ alone were included in parallel. Immediately after the H_2_O_2_ challenge, cells were fixed with immunostaining fixative for 30 min at room temperature and blocked with immunostaining blocking solution. Cells were incubated with the anti-PAR primary antibody for 4 h at room temperature, followed by extensive washing with PBS and incubation with an Alexa Fluor 488-conjugated secondary antibody for 1 h at room temperature. Nuclei were counterstained with DAPI. After washing, fluorescence images were acquired using a Leica SP8 laser confocal microscope, and PAR fluorescence intensities were quantified using image analysis software.

For γ-H2AX immunofluorescence analysis, HCT-15 cells were seeded and cultured under the same conditions and treated directly with P0.7 at 100, 200, or 400 μg/mL for 48 h without H_2_O_2_ stimulation. Cells were fixed with 4% paraformaldehyde for 30 min at room temperature, permeabilized with 0.2% Triton X-100 in PBS for 15 min, and blocked with 5% bovine serum albumin for 1 h. Cells were incubated with the anti-γ-H2AX primary antibody overnight at 4 °C, followed by an Alexa Fluor 488-conjugated secondary antibody for 1 h at room temperature in the dark. Nuclei were counterstained with DAPI. Fluorescence images were acquired using a Leica SP8 laser confocal microscope, and γ-H2AX fluorescence intensities were quantified using image analysis software [37].

### 4.14. In Vivo Antitumor Assay

Six-week-old BALB/c nude mice were acclimatized for 1 week before the experiment at the Medical Laboratory Animal Center of Shandong Second Medical University. All animal procedures were conducted in accordance with institutional guidelines and approved by the Animal Ethics Committee of Shandong Second Medical University (No. 2023SDL050). HCT-15 cells in the logarithmic growth phase were harvested, washed with PBS, and resuspended at an appropriate concentration. Each mouse was inoculated subcutaneously in the right axilla with 5 × 10^6^ HCT-15 cells in 100 μL PBS. After tumors were established, mice were randomly allocated to three groups (*n* = 6 per group): model group, low-dose P0.7 group, and high-dose P0.7 group. The doses of 50 and 100 mg/kg were selected with reference to dose ranges used in previous in vivo antitumor studies of natural polysaccharides [38,39]. Mice in the treatment groups received P0.7 by intraperitoneal injection at 50 or 100 mg/kg once daily for 14 consecutive days, while the model group received an equal volume of saline. Body weight and tumor dimensions were recorded during treatment. At the end of treatment, mice were euthanized, and tumors were excised, weighed, photographed, fixed, and processed for histological and immunohistochemical analyses.

### 4.15. H&E and TUNEL Staining

Tumor tissues were fixed in 4% paraformaldehyde, dehydrated, embedded in paraffin, and sectioned at 4 μm. For H&E staining, sections were deparaffinized, rehydrated, stained with hematoxylin, differentiated, blued, and counterstained with eosin. After dehydration, clearing, and mounting, tissue morphology was examined using a light microscope. For TUNEL staining, paraffin sections were processed using a commercial TUNEL kit according to the manufacturer’s instructions. TUNEL-positive signals were observed and imaged using a microscope [40].

### 4.16. Immunohistochemical Analysis

Paraffin-embedded tumor sections were deparaffinized and rehydrated. Endogenous peroxidase activity was blocked with 3% hydrogen peroxide at room temperature for 10 min. Antigen retrieval was performed using sodium citrate buffer. After cooling and washing with PBS, sections were blocked with 10% normal goat serum and incubated overnight at 4 °C with primary antibodies against Ki67 or γ-H2AX. Sections were then washed and incubated with an HRP-conjugated secondary antibody at 37 °C for 30 min. DAB was used for chromogenic development, and nuclei were counterstained with hematoxylin. After dehydration, clearing, and mounting, positive staining was examined and photographed using a light microscope.

### 4.17. Statistical Analysis

Data are presented as mean ± standard deviation (SD). In vitro experiments were independently repeated three times. Comparisons among multiple groups were conducted by one-way analysis of variance followed by Tukey’s post hoc test. Tumor volume and body weight data were analyzed by repeated-measures analysis of variance followed by appropriate multiple comparisons testing. A value of *p* < 0.05 was considered statistically significant. Fluorescence intensity, positive staining signals, and Western blot band intensities were quantified using ImageJ software.

## Data Availability

Data are contained within the article. The original contributions presented in this study are included in the article. Further inquiries can be directed to the corresponding author.
